# Lili-Hics trial: Efficacy of the lipid test in liver hydatid cyst surgery

**DOI:** 10.17305/bb.2024.10371

**Published:** 2024-10-01

**Authors:** Mehmet Zafer Sabuncuoglu, Isa Sozen, Ismail Zihni, Girayhan Celik, Ayfer Sen Acar, Fatmanur Dal, Demet Gunduz

**Affiliations:** 1 Hepatopancreatobiliary and Liver Transplantation Unit, Department of General Surgery, Faculty of Medicine, Suleyman Demirel University, Isparta, Turkey; 2 Department of Biostatistic and Medical Informatics, Faculty of Medicine, Suleyman Demirel University, Isparta, Turkey; 3 Department of Radiology, Faculty of Medicine, Suleyman Demirel University, Isparta, Turkey

**Keywords:** Bile leak, transcystic lipid injection, hepatic resection, hydatid cyst

## Abstract

Bile leakage is a common complication following liver surgeries, particularly in the cases of liver hydatid cyst operations. Currently, there is no adequate method which could be utilized to prevent this complication effectively. Our study aimed to assess the efficacy of the biliary lipid test (BLT) in reducing biliary complications after hydatid cyst surgery. We retrospectively included patients who underwent open liver hydatid cyst surgery between January 2011 and January 2024. The study encompassed 122 patients, with 41 males and 81 females, ranging in age from 18 to 79 years. In the BLT group, a lipid solution was injected transcystically after cholecystectomy. The BLT was performed on 65 patients, while 57 patients did not undergo the test. Following the transcystic injection of the lipid solution, orifices at the site of lipid droplets that became visible were ligated with 5.0 prolene sutures. A total of 200 leak sites were sutured. Remarkably, none of the patients in the BLT group experienced postoperative bile leakage lasting more than 1 week. Consequently, a shorter hospital stay was observed in this group. Transcystic injection of the lipid solution with distal clamping effectively demonstrated leak sites, and suturing these sites prevented postoperative bile leakage. Our study demonstrates the effectiveness of the LIpid test in LIver Hydatid Cyst Surgery (Lili-Hics) in reducing biliary complications following hydatid cyst surgery.

## Introduction

Biliary fistulas (BFs) represent the predominant cause of postoperative morbidity following hepatic cyst surgery, potentially leading to peritonitis, sepsis, and even postoperative mortality [1]. It is often challenging to detect the occult cysto-biliary communication (CBC) during surgery. The efficacy of emergency surgery for these patients, especially in the absence of endoscopic therapy or its failure, remains unclear. Intraoperative BF tests (BFTs) are considered to be effective in minimizing postoperative BF following hepatic resections [2]. Various agents have been employed for intraoperative BF detection, each with advantages and limitations affecting their utility [3]. The use of transductal isotonic sodium chloride injection has not been statistically proven effective, owing to the solution’s transparency and inadequacy in identifying minor leakages. Dye tests, such as methylene blue and indocyanine green, are more successful in detecting small leakages. However, the irreversible tissue staining, the limitation of single-use, and the risk of allergic reactions limit their usage [4]. The most recent BFT, using a diluted fat emulsion known as biliary lipid test (BLT), is considered to be more applicable. This is because the white-colored lipid droplets are easily visible on the resection surface and can be applied multiple times [3, 5].

The primary cause of morbidity following hepatic hydatid cyst surgery is postoperative BF, which is often asymptomatic preoperatively. Literature indicates that this complication occurs in 8%–26% of patients [6].

While BLTs are utilized in hepatic resection surgeries, such as segmental hepatic resections or malignancy surgeries, there are no documented instances of BLTs being used in liver hydatid cyst surgeries in the literature. For a long time, we have employed the bile leakage test as a standard method to identify potential bile leaks in elective surgery for liver hydatid cysts. The aim of this study is to investigate the outcomes of intraoperative use of the BLT in hydatid cyst surgery and its impact on the incidence of postoperative BF.

## Materials and methods

### Study design and measured outcomes

Patients deemed suitable for surgery were operated on by two experienced surgeons. The patient selection was not randomized. One surgeon performed the BLT, while the other relied on the conventional method with direct visual inspection.

For both patient groups, factors, such as age, gender, cyst count, cyst size, cyst location, and whether the cysts were primary or recurrent were analyzed. In the BLT group, all visible orifices identified by lipid leakage were obliterated. Postoperative bile drainage volumes were recorded in the patients’ files, and drains were removed once no further drainage was observed. Drainage persisting for less than 10 days was classified as temporary bile leakage, whereas drainage extending beyond 10 days was categorized as a BF. Furthermore, within the BF group, patients with less than 100 cc of drainage received conservative management, while those with drainage exceeding 100 cc were treated through endoscopic retrograde cholangiopancreatography (ERCP).

**Figure 1.**
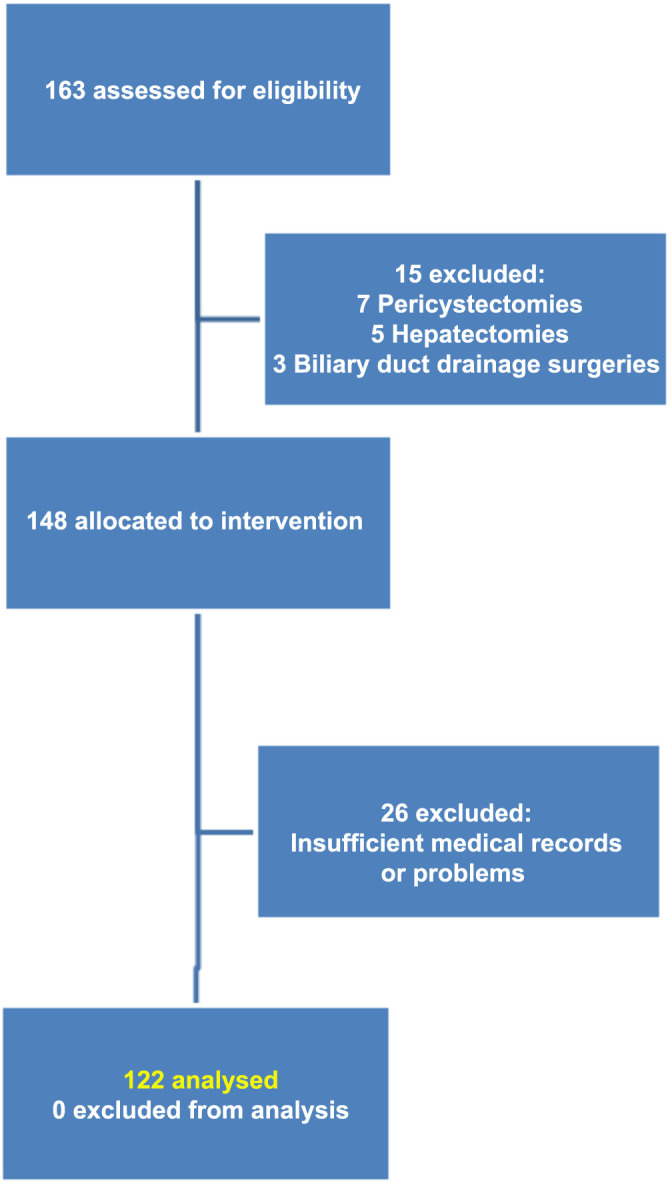
Flow diagram of patient selection and exclusion in this study.

The patients who underwent surgeries, such as pericystectomy, hepatectomy, biliary duct drainage surgeries (T-tube or choledochoduodenostomy), were excluded from the study. The patient selection and exclusion process is illustrated in the flow diagram in Figure 1.

### Medical therapy

To prevent recurrence, all patients received preoperative therapy with albendazole (10 mg/kg/day) for one month. The overall treatment duration continued postoperatively for an average of nine months.

### Surgical technique

The procedure begins with the puncture and aspiration of the cyst, followed by unroofing the cyst. The cyst contents, including the germinative membrane and daughter cysts, are evacuated. At this stage, bile duct orifices are identified and ligated with 5.0 prolene sutures. A drainage catheter is then placed inside the cyst cavity. If no drainage is observed, the drain is removed on the third day. If there is drainage, the drain remains in place until the bile drainage ceases.

**Figure 2.**
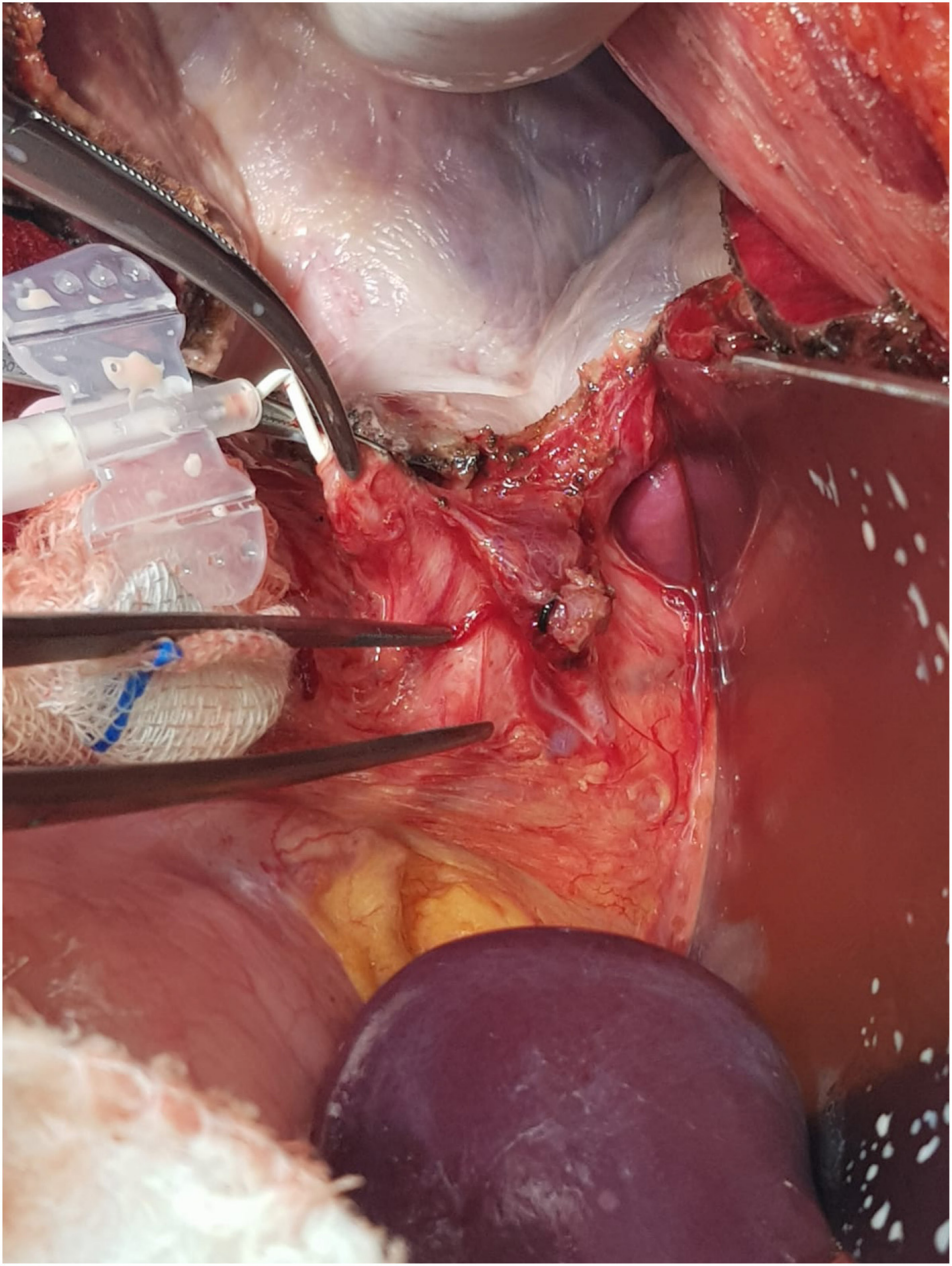
Displaying the insertion of the catheter into the cystic duct.

**Figure 3.**
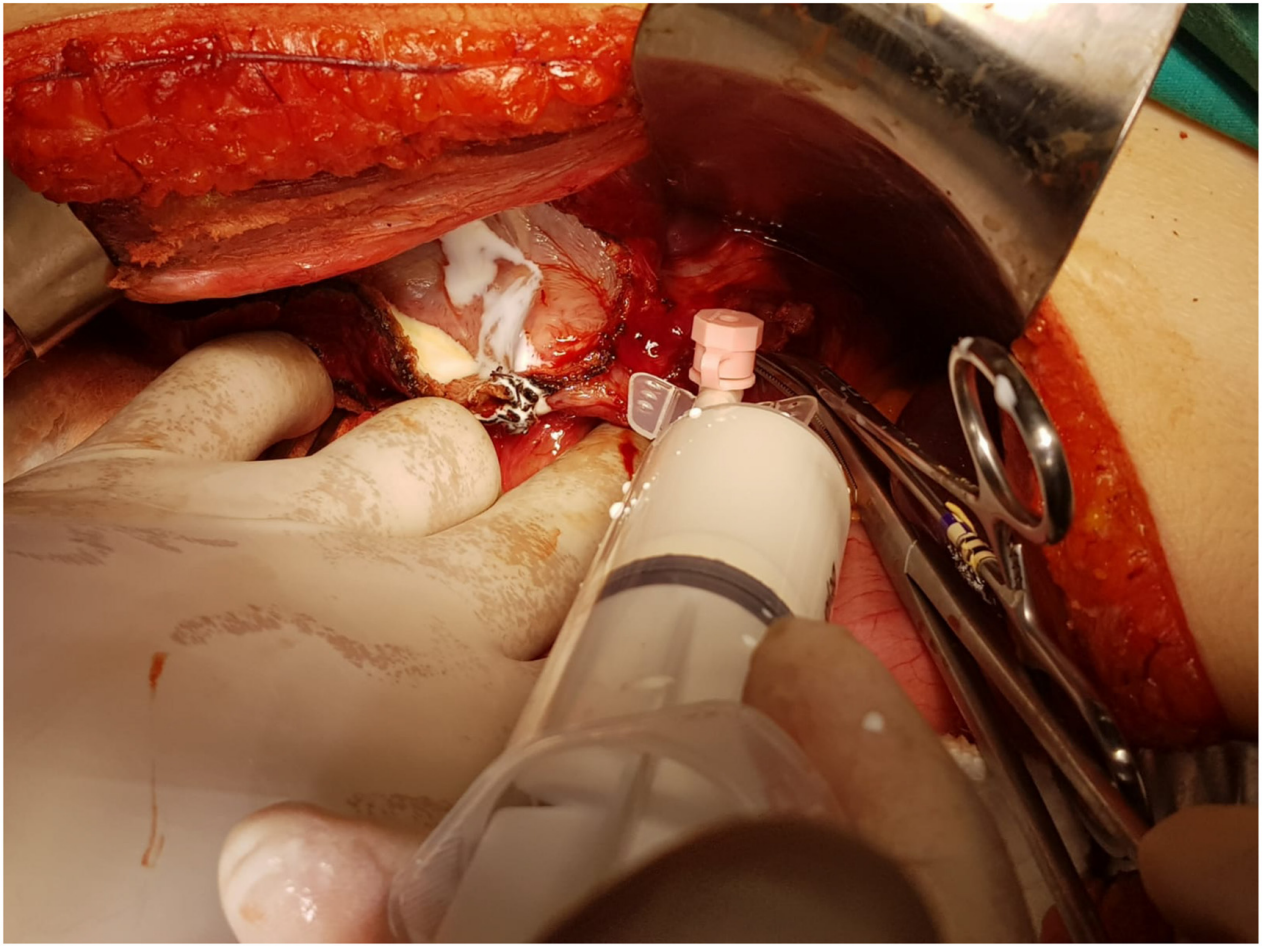
Displaying the orifices at the site of lipid solution injection.

In the BLT group, following cholecystectomy, a 22G catheter was inserted into the cystic duct (Figure 2). Subsequent to the occlusion of the distal portion of the common bile duct, the lipid component of the total parenteral nutrition solution was slowly injected. Orifices where lipid solution droplets became visible were ligated with 5.0 prolene sutures (Figure 3). This procedure was repeated until no lipid leakage was observed (Video 1). Afterward, the catheter was removed, and the cystic duct was ligated. Placement of the drainage catheter was carried out in the same manner as in the group not subjected to the BLT.

### Ethical statement

The study plan was evaluated and approved by the Institutional Ethics Committee of our University Hospital. The approval from the local Ethics Committee was granted under reference number 160, dated August 16, 2023. All procedures were conducted in accordance with the Helsinki Declaration of 1964 and its subsequent versions. All patients had been previously informed, and written consent was obtained from each patient before surgery, indicating that their data could be utilized for scientific research in the future. This is pertinent as the current study is a retrospective study of patients who underwent hepatic cyst surgery employing either intraoperative BLT or the conventional method with direct visual inspection. These procedures were carried out in the General Surgery Department of Suleyman Demirel University Hospital, Faculty of Medicine, Isparta, between January 2011 and July 2024.

### Statistical analysis

The hydatid cyst dataset, comprising 122 observations, is structured around both categorical and continuous variables. This analysis considers various patient characteristics, utilizing multiple statistical methods. The “Day of drain removal” variable was analyzed both numerically and by categorization into two groups: less than 10 days and more than 10 days. Additionally, the “Number of orifices” variable was segmented into five groups. The dataset is noted for its completeness, with no missing values reported.

Data were imported into SPSS 23 (IBM Inc., Chicago, IL, USA) for statistical analysis. Preliminary checks ensured accuracy in data entry and the appropriateness of parameter ranges. The normality of continuous variables was assessed using the Shapiro–Wilk test. Descriptive statistics for continuous variables are presented as mean and standard deviation, whereas categorical variables are described through frequency (*n*) and percentage (%). The Independent Samples Median Test was applied for two-group comparisons in non-normally distributed data. Where relationships were identified, the receiver operating characteristic (ROC) analysis was conducted for continuous variables. The association between categorical variables was examined using the chi-square test analysis. Throughout the analyses, a *P* value of < 0.05 was considered statistically significant.

## Results

Between January 2011 and January 2024, 122 patients underwent hydatid cyst surgery, with a total of 194 cysts being treated. Among these patients, 41 were female and 81 were male. The mean age of the patients was 47.61 ± 14.88 years, with a range from 18 to 79 years. The distribution of cyst numbers per patient was as follows: 76 patients had one cyst, 28 had two cysts, 12 had three cysts, 4 had four cysts, and 2 had five cysts. The mean cyst diameter was 86.86 ± 35.65 mm (ranging from 20 to 210 mm). In terms of cyst location, the largest cyst was found in the medial segments of the liver in 57 patients and in the lateral segments in 65 patients. Preoperatively, 24 patients exhibited elevated liver function test (LFT) values. The BLT was performed on 65 patients, while 57 patients did not undergo the test. In the BLT group, a total of 137 orifices were obliterated, compared to 63 orifices in the group without the test. Postoperative biliary complications were observed in 4 patients from the BLT group and in 3 patients from the group that did not receive the test.

As presented in Table 1, the shortest duration for drain removal was 2 days, and the longest was 27 days, with the mean day of drain removal being 5.97 days with a standard deviation of 4.09. The minimum postoperative hospital discharge (PHT) was 3 days, whereas the maximum was 29 days. The mean PHT was calculated at 7.12 days, with a standard deviation of 4.41. Cyst diameters ranged from a minimum of 20 mm to a maximum of 210 mm, with a mean diameter of 86.86 mm and a standard deviation of 35.65. The youngest patient in the data was 18 years old, while the oldest patient was 79 years old. The mean age of the patients was 47.61 with a standard deviation of 14.87. The analysis concluded that the age of the patients does not significantly influence the application of the BLT (*P* > 0.05).

**Table 1:** Summary of clinical parameters including day of drain removal, postoperative hospital discharge, cyst diameter, and age

**Variables**	**Median (Range)**	**Mean ± SD**	***P***
Day of drain removal	5 (2–27)	5.97 ± 4.09	<0.001*
Postoperative hospital discharge	6 (3–29)	7.12 ± 4.41	<0.001*
Cyst diameter	80 (20–210)	86.86 ± 35.65	<0.001*
Age	50 (18–79)	47.61 ± 14.87	0.012

*Indicating a significant relationship between the variables (*P* < 0.05). SD: Standard deviation.

The average day of drain removal was 4.66 days, with a median of 4 days (ranging from 2 to 10 days) in the BLT group, compared to 7.47 days, with a median of 6 days (ranging from 2 to 27 days) in the no-BLT group. The median drain removal day in the BLT group was significantly lower than that in the no-BLT group (*P* < 0.05). The average day of discharge was 5.67 days, with a median of 5 days (ranging from 3 to 11 days) in the BLT group, and 8.77 days, with a median of 7 days (ranging from 3 to 29 days) in the no-BLT group. The median day of discharge in the BLT group was significantly lower than that in the no-BLT group (*P* < 0.05).

**Figure 4.**
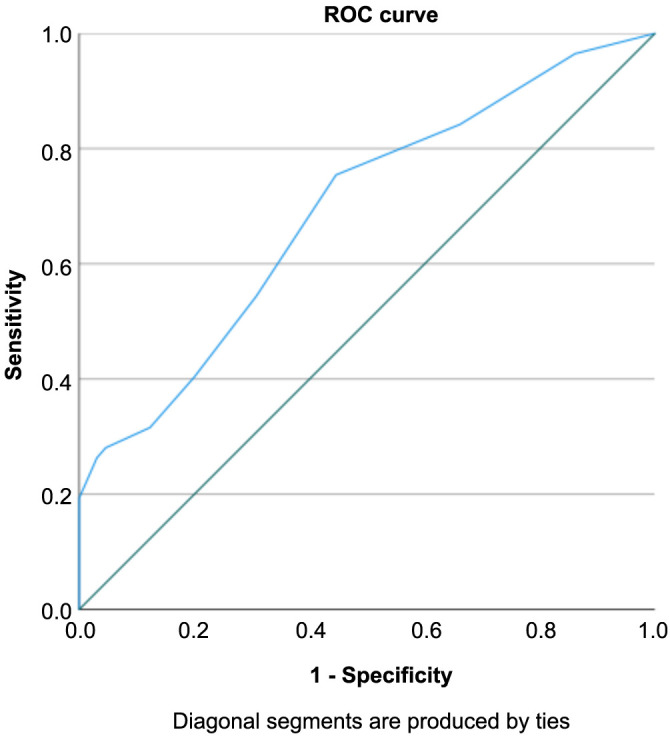
**ROC curve for PHT analysis.** ROC: Receiver operating characteristic; PHT: Postoperative hospital discharge.

In the BLT group, the area under the ROC curve for PHT was 0.69, with a standard error of 0.048. The cut-off value of 5.50 (sensitivity ═ 0.754; specificity ═ 0.554) suggests that patients who underwent the BLT may experience a reduction of 5.50 days in hospital stay (Figure 4).

Temporary bile leakage was observed in seven patients from the BLT group as opposed to nine from the no-BLT group. Notably, BFs were observed in ten patients from the no-BLT group, whereas no cases of BF were reported in the BLT group (*P* < 0.001). There were eight patients with drainage exceeding 10 days and more than 100 cc, and another eight patients with drainage for less than 10 days but also over 100 cc. Following surgery, seven patients in the BLT group and nine in the no-BLT group required ERCP treatment (Table 2).

As seen in Table 2, the BLT was performed on 36 female patients (44.4%) and 20 male patients (48.8%), while 45 female patients (55.6%) and 21 male patients (51.2%) did not undergo any testing. There was no significant association between sex, cyst type, cyst count, and the application of the BLT (*P* > 0.05). Furthermore, in the BLT group, seven patients (43.8%) required postoperative ERCP, compared to nine patients (56.3%) in the group that did not undergo the test. No significant difference was observed in the requirement for ERCP between the two groups (*P* > 0.05).

**Table 2:** Comparison of clinical parameters between the BLT group and the no-BLT group

**Variables**	**Categories**	**BLT group**	**No-BLT group**	***P***
*Sex*				
	Female	36 (44.4)	45 (55.6)	0.479
	Male	20 (48.8)	21 (51.2)	
*Number of cysts*				
	1	38 (50)	38 (50)	0.201
	2	13 (46.4)	15 (53.6)	
	3	10 (83.3)	2 (16.7)	
	4	3 (75.0)	1 (25.0)	
	5	1 (50.0)	1 (50.0)	
*Cyst type*				
	1	6 (50.0)	6 (50.0)	0.421
	2	10 (55.6)	8 (44.4)	
	3	35 (58.3)	25 (41.7)	
	4	13 (50.0)	13 (50.0)	
	5	1 (16.7)	5 (83.3)	
*ERC*				
	Postop	7 (43.8)	9 (56.3)	0.333
	None	57 (55.9)	45 (44.1)	
	Preop	3 (75.0)	1 (25.0)	
*Leakage*				
	Yes	13 (37.1)	22 (62.9)	0.023*
	None	52 (59.8)	35 (40.2)	
*Preop LFT*				
	Normal	52 (53.1)	46 (46.9)	0.923
	High	13 (54.2)	11 (45.8)	
*Previous operations*				
	Yes	25 (53.2)	22 (46.8)	0.947
	None	31 (52.5)	28 (47.5)	
*Orifice obliteration*				
	Yes	42 (62.7)	25 (37.3)	0.022*
	None	23 (41.8)	32 (58.2)	
*Orifice count*				
	1	9 (69.2)	4 (30.8)	0.192
	2–3	17 (50.0)	17 (50.0)	
	4–5	10 (71.4)	4 (28.6)	
	6–7	4 (100.0)	0 (0.0)	
	8–10	2 (100.0)	0 (0.0)	
*Bile drainage over 100 cc*				
	Yes	7 (43.8)	9 (56.3)	0.412
	None	58 (54.7)	48 (45.3)	
*Day of drain removal*				
	Lower than 10	65 (58.0)	47 (42.0)	<0.001*
	Greater than 10	0 (0.0)	10 (100.0)	
*Recurrence*				
	Yes	22 (47.8)	24 (52.2)	0.348
	None	43 (56.6)	33 (43.4)	

Regarding the cyst type, the Gharbi hydatid cyst classification was used. *Indicating a significant relationship between the variables (*P* < 0.05). The numbers represent *n* (%). BLT: Biliary lipid test; ERC: Endoscopic retrograde cholangiopancreatography; Postop: Postoperative; Preop: Preoperative; LFT: Liver function test.

BF was observed in 13 patients (37.1%) upon whom the BLT was performed and in 22 patients (62.9%) who did not undergo the test. In the BLT group, 52 patients (59.8%) experienced no leakage, demonstrating a statistically significant correlation between the incidence of leakage and the administration of the BLT (*P* < 0.05) (Table 3). Patients in both groups were categorized according to the number of orifices obliterated, with groupings as follows: 1, 2–3, 4–5, 6–7, and 8–10 orifices. There was no statistically significant association between the count of obliterated orifices and the application of the BLT (*P* > 0.05). Additionally, in the BLT group, seven patients (43.8%) had bile drainage exceeding 100 cc, compared to nine patients (56.3%) in the no-BLT group. All patients requiring drainage for more than 10 days were from the no-BLT group, while 65 patients (58.0%) whose drains were removed in less than 10 days had undergone the BLT (*P* < 0.05) (Table 2).

Table 4 indicates that there is a significant relationship between the necessity for postoperative ERCP and the occurrence of leakage when comparing the BLT and no-BLT groups (*P* < 0.001). There is no significant association between preoperative LFT, the count of orifices, recurrence, and the occurrence of leakage (*P* > 0.05). While in the group that did not undergo the BLT, a correlation was found between orifice obliteration and leakage, this correlation was not observed in the BLT group. Furthermore, in the BLT group, no patients who experienced leakage had drainage for more than 10 days.

**Table 3:** Contingency table of categorical variables stratified by leakage status

**Variables**	**Categories**	**Leakage**	**No leakage**	***P***
*Day of drain removal*				
	Lower than 10	26 (23.2)	86 (76.8)	<0.001*
	Greater than 10	9 (90.0)	1 (10)	
*Orifice count*				
	1	5 (38.5)	8 (61.5)	0.164
	2–3	17 (50.0)	17 (50.0)	
	4–5	3 (21.4)	11 (78.6)	
	6–7	0 (0.0)	4 (100.0)	
	8–10	1 (50.0)	1 (50.0)	
*Preop LFT*				
	Normal	27 (27.6)	71 (72.4)	0.575
	High	8 (33.3)	16 (66.7)	
*Orifice obliteration*				
	Yes	26 (38.8)	41 (61.2)	0.006*
	None	9 (16.4)	46 (83.6)	
*Recurrence*				
	Yes	13 (28.3)	33 (71.7)	0.935
	None	22 (28.9)	54 (71.1)	
*Bile drainage over 100 cc*				
	Yes	14 (87.5)	2 (12.5)	<0.001*
	None	21 (19.8)	85 (80.2)	
*ERC*				
	Postop	16 (100.0)	0 (0.0)	<0.001*
	None	16 (15.7)	86 (84.3)	
	Preop	3 (75.0)	1 (25.0)	

*Indicating a significant relationship between the variables (*P* < 0.05). The numbers represent *n* (%). LFT: Liver function test; Preop: Preoperative; ERC: Endoscopic retrograde cholangiopancreatography; Postop: Postoperative.

**Table 4:** Comparison of leakage between the BLT group and the no-BLT group

		**BLT group (*n* ═ 65)**			**No-BLT group (*n* ═ 57)**		
**Variables**	**Categories**	**Leakage**	**No leakage**	***P***	**Leakage**	**No leakage**	***P***
*ERC*							
	Postop	7 (100.0)	0 (0.0)	<0.001*	9 (100.0)	0 (0.0)	<0.001*
	None	5 (8.8)	52 (91.2)		11 (66.7)	34 (75.6)	
	Preop	1 (100.0)	0 (0.0)		2 (66.7)	1 (33.3)	
*Preop LFT*							
	Normal	9 (17.3)	43 (82.7)	<0.274	18 (39.1)	28 (60.9)	0.865
	High	4 (30.8)	9 (69.2)		4 (36.4)	7 (63.6)	
*Orifice obliteration*							
	Yes	11 (26.2)	31 (73.8)	0.115	15 (60.0)	10 (40.0)	0.003
	None	2 (8.7)	21 (91.3)		7 (21.9)	25 (78.1)	
*Orifice count*							
	1	4 (44.4)	5 (55.6)	0.287	1 (25.0)	3 (75.0)	0.338
	2–3	5 (29.4)	12 (70.6)		12 (70.6)	5 (29.4)	
	4–5	1 (10.0)	9 (90.0)		2 (50.0)	2 (50.9)	
	6–7	0 (0.0)	4 (100.0)		0 (0.0)	0 (0.0)	
	8–10	1 (50.0)	1 (50.0)		0 (0.0)	0 (0.0)	
*Bile drainage over 100 cc*							
	Yes	6 (85.7)	1 (14.3)	<0.001*	8 (88.9)	1 (11.1)	<0.001*
	None	7 (12.1)	51 (87.9)		14 (29.2)	34 (70.8)	
*Day of drain removal*							
	Lower than 10	13 (20.0)	52 (80.0)		13 (27.7)	34 (72.3)	<0.001*
	Greater than 10	0 (0.0)	0 (0.0)		9 (90.0)	1 (10.0)	
*Recurrence*							
	Yes	1 (4.5)	21 (95.5)	0.057	12 (50.0)	12 (50.0)	0.132
	None	12 (27.9)	31 (72.1)		10 (30.3)	23 (69.7)	

*Indicating a significant relationship between the variables (*P* < 0.05). The numbers represent *n* (%). BLT: Biliary lipid test; ERC: Endoscopic retrograde cholangiopancreatography; Postop: Postoperative; Preop: Preoperative; LFT: Liver function test.

## Discussion

Hydatid cyst disease of the liver can be treated with a variety of surgical interventions, from simple cyst drainage to major hepatic resection. Despite the benign nature of the disease, its outcomes and characteristics may pose serious health risks and require appropriate treatment.

Surgical preferences vary among clinicians. Some opt for extensive procedures like hepatectomy, while others favor conservative approaches. In cases where anatomical considerations render surgery impractical, conservative management might be preferred. Particularly following conservative surgical interventions, connections between the cyst and biliary ducts can lead to numerous complications [7, 8]. To mitigate the incidence of postoperative BF, techniques, such as T-tube placement, cystoenteric anastomosis, and fibrin glue application, have been employed [9].

The incidence of BF and cavity-related complications remains higher after conservative procedures, largely due to the presence of undetectable bile duct orifices within the hydatid cyst cavity. Postoperative transient BF or persistent BF occurs in 8.2%–26% of patients undergoing conservative surgery [10].

Despite the application of these techniques, the incidence of postoperative fistulas has not been reduced to less than 14%. Solely performing biliary decompression has also failed to sufficiently diminish complication rates. However, suturing of biliary orifices combined with T-tube placement has proven more effective in reducing the occurrence of BF.

ERCP is most commonly used for patients with obstructive jaundice or acute cholangitis to alleviate bile duct obstruction. ERCP may reveal daughter cysts within the duodenum, at the ampulla of Vater, or obstructing parts of the biliary tree. The overall success rate of this procedure is between 70% and 86%, with a fistula closure rate of 81% within 1–2 days. For fistulas smaller than 5 mm, suturing over healthy tissue post-capsule removal, along with adequate drainage, is a viable treatment. However, this technique’s primary disadvantage is the potential for prolonged BF and the formation of subphrenic abscesses [11].

Routine ERCP is not yet universally accepted for uncomplicated hydatid cysts. However, some clinical centers recommend its use to thoroughly examine bile duct anatomy and delineate the communication between the cyst and bile ducts. Yet, ERCP may not always detect subtle communications and can lead to the formation of postoperative BF. Even when conducted by highly skilled practitioners, ERCP can have severe complications, such as pancreatitis, bleeding, infection, or perforation [6, 12]. Preoperatively identifying CBC, along with performing sphincterotomy and ERCP, can decrease the risk of postoperative bile leakages. Given its invasive nature, ERCP should only be carried out by experienced personnel. However, biliary decompression alone, without disconnecting the cyst from the biliary system, has not been shown to effectively reduce the incidence of BF or related complications [10]. BF tends to close more slowly when the cysto-biliary defect is large, located in the main biliary tree, and accompanied by a high flow of bile [6].

In our study, the occurrence rate of BF after implementing the BLT without T-tube administration was found to be 0%. Due to the risk of stenosis and associated complications in the biliary duct, we decided to not use the T-tube placement technique. While omentoplasty to the cyst cavity could be an effective technique, complication rates have been reported to range from 13% to 89% [13]. Simply filling the cavity with omentum without securing the biliary orifices does not entirely preclude the risk of BF or cavity infections [10].

Particularly for cysts situated at the liver apex, omentoplasty can prevent imaging findings that might be mistaken for recurrence. In our procedure, following the BLT, omentoplasty was performed in all cases where the omentum was deemed suitable. However, in patients with multiple cysts, prior omentectomy, or previous surgeries, the applicability of omentoplasty is limited.

The use of fibrin glue in the cyst cavity has been proposed as a technique, but given that hydatid disease is more prevalent in developing countries, fibrin glue administration can be considered relatively expensive. Additionally, the use of fibrin glue following hepatectomy did not demonstrate a reduction in biliary complication rates compared to prior studies. An overall 40% complication rate was noted [14]. Thus, the application of fibrin glue on the hepatectomy surface is not supported as an effective measure to prevent biliary complications [10].

Vreeland et al. [15] recommended the air leak test as a feasible method, but note its limitations in patients who have not undergone diaphragm resection, as the air bubble test may not reveal all orifices. Shimizu et al. [16] also recommended an intraoperative air leak test through air injection into the bile duct and obliteration of small biliary orifices. However, the validity of the results could be questionable due to the small sample size of the study.

Trehan et al. suggested that a diluted 1.5% H_2_O_2_ test may be more effective than the conventional saline test in detecting bile leaks during liver resection and donor hepatectomies, as evidenced in their series of 31 patients. While H_2_O_2_ shows promising results, its long-term effects on the bile duct remain uncertain [17]. Recent advancements in liver and biliary tract surgery have decreased the need for potentially toxic procedures, such as this one, for the bile ducts [11].

Sakaguchi et al. in a how I do it article, recommended an intrabiliary injection of indocyanine green solution through a transcystic tube with distal common bile duct clamping, using an infrared camera system to visualize the leaks. Although no postoperative leaks were found in 27 patients who underwent hepatic resection using this method, the expense and the need for specific equipment could restrict its application in routine practice [18].

Tanaka et al. highlighted that during conventional leak tests, it can be challenging to ensure that the testing reagent sufficiently reaches the intrahepatic bile duct near the liver’s cut surface with adequate volume and pressure to identify bile leaks. They proposed conducting the leak test with contrast-enhanced intraoperative ultrasonic cholangiography (CE-IOUSC), utilizing Sonazoid as a contrast agent to enhance the visualization of dye injection into the intrahepatic bile duct [19].

Diana et al. introduced a novel laparoscopic technique using narrow-band imaging for real-time detection of bile leaks during hepatectomy in a porcine model. This technique, utilizing the SPECTRA-A device from Karl Storz in Tuttlingen, Germany, enables the direct visualization of bile leakage without the need for dye injection. However, its implementation is limited by the need for specialized equipment, which may not be available in many centers. Despite this, the method shows particular promise for laparoscopic surgeries, where dye or lipid administration poses challenges [20]. This approach might explain occurrences of bile leakage in patients undergoing the BLT. It is noted that almost all hydatid liver cysts can be treated with conservative surgical approaches, which can be executed by a general surgeon using standard surgical equipment [10].

Leelawat et al. conducted a comparison between conventional saline injection and the BLT in 30 patients undergoing elective liver resection. The incidence of postoperative bile leakage was statistically lower in the BLT group. These findings across these various studies underscore the significance of applying leakage tests to significantly reduce bile leakage rates. Nonetheless, there remains a demand for a method that is safe, effective, and economically feasible for daily practice. In this context, a readily available test employing a lipid solution, which is accessible in virtually every clinic, offers a highly effective and low-cost option with minimal side effects [21].

Another study utilizing the saline test also reported a reduced incidence of fistulas among the test group. In this research, the application of the methylene blue leak test revealed that methylene blue can effectively cover the cyst cavity, particularly in cases where multiple orifices exist within the same cavity. However, methylene blue is not an easily removable dye, complicating the identification of additional orifices once the cyst is coated with the dye [22].

Furthermore, the complications arising from hydatid cyst disease must be accurately diagnosed, evaluated, and treated. The most common complications include infection, intrabiliary rupture, intrathoracic rupture, and rupture into the peritoneal cavity. However, rarer complications, such as portal hypertension and even fistulization into the skin, may also be observed [23].

Li et al. demonstrated that the BLT conducted during major liver resection surgeries between June 2005 and June 2007 resulted in statistically significant better outcomes compared to a no-BLT group, with bile leakage rates of 5.3% vs 22.9%, respectively. Given that the study included 137 patients in a prospective cohort design and that leakages were assessed on the 30th postoperative day, it was suggested that the BLT could become a standardized method for preventing bile leakage [2]. This method is deemed safe enough for use in donors and is associated with minimal morbidity.

This testing method can be universally applied to all cases of hydatid liver cysts without any specific selection criteria and can be used in combination with other methods, such as omentoplasty and T-tube drainage, as needed [10]. Although liver resection is rarely mentioned in the literature for treating liver hydatid cysts, these approaches typically require a surgeon with expertise in liver resections and might sometimes need specialized surgical equipment. Radical techniques that ensure the closure of CBC in healthy tissue have been shown to reduce postoperative morbidity [11].

## Conclusion

Our study aimed to evaluate the effectiveness of the BLT in reducing biliary complications following hydatid cyst surgery. Due to its repeatability and superior visualization capabilities, the BLT emerges as a more effective approach. Importantly, it can be conducted by a general surgeon using standard surgical equipment, making it a practical option for widespread adoption.

## Supplemental data

**VIDEO 1.** **Illustrating the lipid solution leakage through an orifice**

The video is available at the following link:

https://www.bjbms.org/ojs/index.php/bjbms/article/view/10371/3454

## Data Availability

The data supporting the findings of this study are available from the corresponding author upon reasonable request.
